# Effects of Repetition Suppression on Sound Induced Flash Illusion With Aging

**DOI:** 10.3389/fpsyg.2020.00216

**Published:** 2020-02-21

**Authors:** Yawen Sun, Xiaole Liu, Biqin Li, Clara Sava-Segal, Aijun Wang, Ming Zhang

**Affiliations:** ^1^Department of Psychology, Soochow University, Suzhou, China; ^2^Research Center for Psychology and Behavioral Sciences, Soochow University, Suzhou, China; ^3^Laboratory of Psychology and Cognition Science, School of Psychology, Jiangxi Normal University, Nanchang, China; ^4^Department of Neurology & Neurological Sciences, Stanford University, Palo Alto, CA, United States

**Keywords:** repeated auditory stimuli, sound-induced flash illusion, multisensory integration, repetition suppression, fission illusion, fusion illusion

## Abstract

The sound-induced flash illusion (SiFI) is a classical auditory-dominated multisensory integration phenomenon in which the observer misperceives the number of visual flashes due to the simultaneous presentation of a different number of auditory beeps. Although the SiFI has been documented to correlate with perceptual sensitivity, to date there is no consensus as to how it corresponds to sensitivity with aging. The present study was based on the SiFI paradigm (Shams et al., 2000), adding repeated auditory stimuli prior to the appearance of audiovisual stimuli to investigate the effects of repetition suppression (RS) on the SiFI with aging. The repeated auditory stimuli consisted of one or two of the same auditory stimuli presented twice in succession, which were then followed by the audiovisual stimuli. By comparing the illusions in old and young adults, we aimed to explore the influence of aging on the RS of auditory stimuli on the SiFI. The results showed that both age groups showed SiFI effects, however, the RS performance of the two age groups had different effects on the fusion and fission illusions. The illusion effect in old adults was weaker than in young adults. Specifically, RS only affected fission illusions in the old adults but both fission and fusion illusions in young adults. Thus, the present study indicated that the decreased perceptual sensitivity based on auditory RS could weaken the SiFI effect in multisensory integration and that old adults are more susceptible to RS, showing that old adults perceived the SiFI effect weakly under auditory RS.

## Introduction

Our bodies receive all kinds of information simultaneously, and most of this information arrives in multisensory modalities. There are two main types of behavioral outcomes to multisensory integration. The first type are multisensory illusion effects, which involve merging of information across senses; in some cases, one sensory modality within the multisensory information competes for preferential access to the consciousness, which means that this modality dominates the others to receive preferential processing during multisensory competition and eventually dominates the awareness and behavior of the observer (Stein and Meredith, 1993; Driver and Noesselt, 2008; Koelewijn et al., 2010; Talsma et al., 2010; Spence, 2011; Chen and Zhou, 2013; Huang et al., 2015).

Visual information dominates other sensory modalities more frequently. However, auditory information can also dominate other sensory modalities, especially when temporal information is involved (Repp, 2000, 2002; Bresciani et al., 2008; Wang et al., 2019). Shams et al. (2000) defined a specific phenomenon of information competition, the sound-induced flash illusion (SiFI). A certain number of visual responses are presented with an unequal number of auditory stimuli that are either presented successively or simultaneously within 100 ms. People will misperceive the number of visual flashes due to the simultaneous presentation of the different numbers of auditory beeps. This effect can be divided into fission and fusion illusions (Shams et al., 2000, 2002; Andersen et al., 2004). The fission illusion occurs when two flashes are perceived if a single flash is accompanied by two auditory stimuli (Shams et al., 2000, 2002); the fusion illusion occurs when one flash is perceived if two flashes are accompanied by one auditory stimulus (Andersen et al., 2004).

Some studies have also suggested that a higher SiFI corresponds to lower sensitivity; a lower perceptual sensitivity, calculated by *d’*, can indicate susceptibility to the illusion (McCormick and Mamassian, 2008; Kumpik et al., 2014). Repetition suppression (RS) is known to influence perceptual sensitivity (Sobotka and Ringo, 1994; Grill-Spector and Malach, 2001); that is, when the same visual stimuli are repeated, the participants had a faster response to the stimuli and a lower error rate (Ferrand and Grainger, 1992; Schacter and Buckner, 1998; James et al., 1999, 2000; Henson, 2003), and the amplitude of neural activity in the cortex induced by this stimulus decreased significantly following presentation of the repeated stimuli (Grill-Spector et al., 1998, 1999). This reduced activity could last from a few milliseconds (Sobotka and Ringo, 1996) to a few minutes (Henson et al., 2000) and up to several days (Turennout et al., 2001), and the RS of different brain regions varies (Barron et al., 2016). This reduced perceptual sensitivity could be the cause of a greater SiFI and act as a measure of susceptibility to the illusions. This, together with the RS, could influence the intensity of neural activity in the perceptual sensitivity. Therefore, based on the classical SiFI paradigm, we aimed to add repeated stimuli of the auditory modality prior to the presentation of audiovisual stimuli to investigate the effects of RS before audiovisual stimuli on the SiFI.

Existing studies have shown that the SiFI varies across individuals (Keil and Senkowski, 2018; Kaiser et al., 2019). Mishra et al. (2007) found that the proportion of SiFIs perceived ranged from 3 to 86% among individuals. Recent studies have also shown that neural oscillations orchestrate the SiFI effect (Lange et al., 2014; Keil and Senkowski, 2018; Kaiser et al., 2019). Moreover, some studies have indicated that perceptual processing changes dramatically with aging (Cabeza et al., 2004; Salat et al., 2009; Grady, 2010). Some studies have shown that old adults have better multisensory integration than young adults. DeLoss et al. (2013) indicated that there was a greater influence of beeps when judging the number of visual flashes in old adults than in young adults. In addition, old adults were susceptible to the fission illusion across a much wider range of temporal asynchronies in the SiFI (Setti et al., 2011; McGovern et al., 2014), presumably due to an enlarged temporal window of integration (TWI) compared to young adults (Laurienti et al., 2006; Peiffer et al., 2007; Diederich et al., 2008; Wu et al., 2012). However, other studies have shown that old adults have less multisensory integration than young adults. One study showed that old adults demonstrated a significantly greater reaction time (RT) benefit when processing concurrent VS coactivation, while young adults demonstrated a significant increase in the magnitude of AV and AS coactivation (Mahoney et al., 2011). Therefore, there is debate about the evidence for audiovisual integration in old adults (DeLoss et al., 2013; McGovern et al., 2014; DeLoss and Andersen, 2015; Chan et al., 2018).

Previous studies have proven that RS is affected by aging. Some studies have suggested that on repeated fMRI, old adults show delayed repetition of visual processing (Chee et al., 2006; Goh et al., 2010). Other studies have shown that responses to repeated auditory stimuli within trials (Fabiani et al., 2006) or across trials (Aine et al., 2005) have less repetitive effects in old adults than young adults. However, previous studies have focused more on the age-related RS difference in unimodal processing (Grady et al., 2011; Braskie et al., 2012; Miyakoshi et al., 2012; Ballesteros et al., 2013; Silvia and Guilherme, 2017), and to date there is no consensus as to the nature of the difference in multisensory processing. Therefore, based on the classical SiFI paradigm, we added repeated auditory stimuli prior to the presentation of audiovisual stimuli to investigate whether the bottom-up factor of RS affected the SiFI with aging. We hypothesized that the RS of auditory stimuli could affect the SiFI and that the RS would affect the magnitude of the SiFI differently between old and young adults.

## Materials and Methods

### Participants

According to the calculation from the G’Power software (GPower_3.1.7), for power = 0.8 and effect size = 0.5, the total sample size should be 19. We asked 32 young adults and 31 old adults to participate in the present experiment. If the participant’s accuracy (ACC) exceeded the average by two standard deviations (SD) under a certain experimental condition, he or she was considered to be unable to identify the stimulus properly, and the data of the participant were rejected. Based on this criterion, six young adults and four old adults were excluded from the experiment. Therefore, ultimately, the young adult group included 26 college students (8 males and 18 females) aged 18–26 years old (mean age = 22); the old adult group included 27 old adults (6 males and 21 females) aged 60–76 years old (mean age = 64). All participants were naive to the experimental procedure and were paid for their participation in the experiment. All participants were rescreened for self-reported eye disease, neurological disorders (e.g., Alzheimer’s disease, Parkinson’s disease, stroke), and any significant hearing loss. All participants gave written informed consent following the standard of the Declaration of Helsinki. The study was approved by the Ethics Committee of the Department of Psychology, Soochow University.

### Stimuli and Apparatus

**FIGURE 1:**
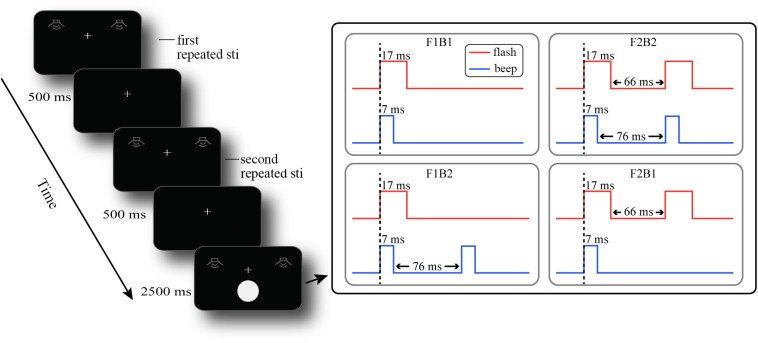
Procedure of events in a sample trial. F1B1 means a visual stimulus accompanied by an auditory stimulus; F1B2 means a visual stimulus accompanied by two auditory stimuli; F2B1 means two visual stimuli accompanied by an auditory stimulus; F2B2 means two auditory stimuli and two visual stimuli.

All stimuli were presented on a View Sonic P220f VS10284 with a screen resolution of 1024 × 768 pixels and a refresh rate of 60 Hz. All visual stimuli in the experiment were presented on a black background by Presentation Software (Neurobehavioral Systems Inc). The visual stimuli were white disks with a radius of view of 2° presented at a 5° viewing angle below the central fixation point. All visual stimuli in the experiment were presented for 17 ms. The visual stimulus was presented 5° below the central fixation point because, with the accompanying auditory stimuli, the visual stimuli have the greatest illusion effect in the peripheral field (Shams et al., 2002; Figure 1). The auditory stimuli in the experiment were presented by a head-mounted iron triangle earphone (ATH-WS99). The auditory stimulus had a loudness of 75 dB, a frequency of 3.5 kHz, and a presentation time of 7 ms (same as the stimuli in Shams et al., 2002).

### Experimental Design and Procedure

The experiment was a 2 (participant group: old vs. young adults) × 2 (number of repeated auditory stimuli: one vs. two) × 2 (number of visual flash stimuli: one vs. two) × 2 (number of auditory stimuli: one vs. two) mixed design; the participant group is the between-group variable, and others are the within-group variables. The latter three factors constituted the eight experimental conditions (A1_F1B1, A1_F1B2, A1_F2B1, A1_F2B2, A2_F1B1, A2_F1B2, A2_F2B1, and A2_F2B2). A1_F2B1 means that there was a repeated auditory stimulus, followed by two visual stimuli accompanied by an auditory stimulus, while A2_F1B2 means that there were two repeated auditory stimuli, followed by a visual stimulus accompanied by two auditory stimuli. We were more interested in the two types of illusion (fission and fusion illusions), so when conducting ANOVA, we integrated the last two variables into one variable: the illusion type (fission illusion: F1B2 vs. fusion illusion: F2B1). At the beginning of the experiment, participants were required to pass a test to determine whether they understood the task and could discriminate the beeps or flashes in isolation. The formal experimental procedure is shown in Figure 1. At the beginning of each trial, the participants were presented with a single auditory stimulus or two consecutive auditory stimuli (repeated auditory stimuli, stimuli duration 7 ms). After a short 500 ms interval, the repeated auditory stimuli were repeated, and the number was the same as before (repeated auditory stimuli). After 500 ms, one or two visual target stimuli (duration 17 ms) accompanied by one or two auditory stimuli (duration 7 ms) were presented, the number of which was independent of the number of previous repeated auditory stimuli. The participants’ task was to determine if they perceived one or two visual stimuli by pressing the left or right mouse button within 2500 ms after the stimuli were presented. We balanced the button response, with half of the participants pressing the left button and half pressing the right button if they perceived one flash. The auditory stimuli were presented simultaneously with the visual stimuli; the interval between the two visual stimuli was 66 ms, and the interval between the two auditory stimuli was 76 ms (right side of Figure 1). The participants were asked to judge the number of visual stimuli and to ignore the auditory stimuli. Each participant needed to complete 480 trials (60 trials per block, 8 blocks in total), 60 trials under each experimental condition, and the interval between trials was randomized from 400 to 700 ms in steps of 100 ms.

## Results

### Comprehensive Analysis of the Two Groups

To explore the effects of RS on SiFI with aging, we performed 2 (participant group: old vs. young adults) × 2 (number of repeated auditory stimuli: one vs. two) × 2 (illusion type: F1B2 vs. F2B1) repeated-measures ANOVA. The main effect of the number of repeated auditory stimuli was significant, *F*(1,51) = 71.19, *p* < 0.001 η*^2^* = 0.58, indicating that the accuracy under two repeated stimuli (83%) was significantly greater than that under one repeated stimulus (75%). The main effect of participant group was significant, *F*(1,51) = 5.99, *p* = 0.018, η*^2^* = 0.11, indicating that the accuracy of the old adults (84%) was significantly greater than that of the young adults (73%). The main effect of illusion type was significant, *F*(1,51) = 4.84, *p* = 0.032, η*^2^* = 0.09, indicating that the accuracy under the fission illusion (82%) was significantly greater than that under the fusion illusion (75%). The interaction between the participant group and the number of repeated auditory stimuli was significant, *F*(1,51) = 12.85, *p* = 0.001, η*^2^* = 0.20, which means that old and young adults showed different performances under one or two repeated stimuli. Simple effect analysis showed that for the one repeated auditory stimulus condition, the accuracy of the old adults (82%) was greater than that of the young adults (68%), *t*(104) = 3.30, *p* = 0.001, Cohen’s *d* = 0.64, *CI* = [5.83, 23.36]. For the two repeated auditory stimuli condition, the accuracy of old adults (87%) was also greater than that of the young adults (79%), *t*(104) = 2.08, *p* = 0.04, Cohen’s *d* = 0.40, *CI* = [0.36, 15.20]. However, there was no significant difference between illusion type and participant group, *F* > 1. There were no significant differences between illusion type and the number of repeated stimuli, *F*(1,51) = 1.12, *p* = 0.29, η*^2^* = 0.02, and there were no significant differences among illusion type, number of repeated auditory stimuli and participant group, *F* < 1. Since there were significant differences in multisensory processing between the old and young adults, we further investigated the potential interaction between illusion type and number of repeated auditory stimuli for the two groups of participants. A 2 (number of repeated auditory stimuli: one vs. two) × 2 (illusion type: F1B2 vs. F2B1) repeated-measures ANOVA was conducted in the old and young adults.

### Old Adult Group Analysis (Mean Age = 64)

To further investigate the potential interaction between illusion type and the number of repeated auditory stimuli in old adults, a 2 (number of repeated auditory stimuli: one vs. two) × 2 (illusion type: fission illusion vs. fusion illusion) repeated-measures ANOVA was conducted for the old adults group. The main effect of the number of repeated auditory stimuli was significant, *F*(1,26) = 17.94, *p* < 0.001, η*^2^* = 0.41, indicating that the accuracy under two repeated stimuli (87%) was significantly greater than that under one repeated stimulus (82%). The main effect of illusion type was significant, *F*(1,26) = 5.81, *p* = 0.02, η*^2^* = 0.18, indicating that the accuracy under fission illusion (88%) was significantly greater than that under fusion illusion (81%). However, the interaction effect between illusion type and the number of repeated auditory stimuli was marginally significant, *F*(1,26) = 3.52, *p* = 0.07, η*^2^* = 0.12, which means that the one and two repeated stimuli conditions showed no significant difference for both fission and fusion illusions. Then, a paired sample *t* test on the accuracy of A1_F1B2 and A2_F1B2 and A1_F2B1 and A2_F2B1 were performed. The results showed that the accuracy of A1_F1B2 (85%) was significantly lower than that of A2_F1B2 (91%), *t*(26) = 4.35, *p* < 0.001, Cohen’s *d* = 0.42, *CI* = [3.46, 9.65]. There was no significant difference in the accuracy between A1_F2B1 (80%) and A2_F2B1 (82%), *t*(26) = 1.78, *p* = 0.087, Cohen’s *d* = 0.15, *CI* = [−0.42, 5.75]. The results suggested that repetition of an auditory-based modality could affect the fission illusion (F1B2), and the accuracy under the one repeated auditory stimulus condition was significantly greater than that of the two repeated auditory stimuli condition. However, the repetition of an auditory-based modality did not affect the fusion illusion (F2B1).

**FIGURE 2:**
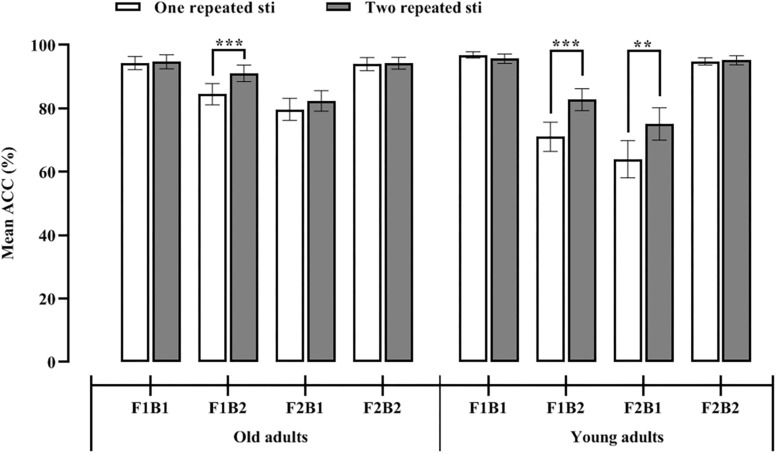
Mean accuracy across all experimental conditions in old **(left)** and young **(right)** adults.

Moreover, whether one or two repeated auditory stimuli was presented, the accuracy of the participants under the F1B1 and F2B2 conditions was significantly higher than that under the other two conditions (see Figure 2); that is, when the number of visual stimuli were consistent with the number of accompanying auditory stimuli, the participants were able to make more accurate judgments that were not affected by the number of repeated stimuli. To prove that the SiFI was present in the old adults, we performed eight paired-sample *t* tests with Bonferroni correction on the following experimental conditions. It can be seen in Figure 2 that the accuracy of the A1_F1B2 condition (85%) was significantly smaller than that of the A1_F1B1 (94%) and A1_F2B2 (94%) conditions [*t*_1_(26) = 3.64, *p_1_* = 0.001 < 0.01/8, Cohen’s *d* = 0.65, *CI* = [4.27, 15.36] and *t*_2_(26) = 3.27, *p_2_* = 0.003 < 0.05/8, Cohen’s *d* = 0.66, *CI* = [3.51, 15.38], respectively]. The accuracy of the A2_F1B2 condition (91%) was lower than that of the A2_F1B1 (95%) and A2_F2B2 (94%) conditions [*t*_1_(26) = 2.63, *p*_1_ = 0.014 > 0.05/8, Cohen’s *d* = 0.29, *CI* = [0.81, 6.53] and *t*_2_(26) = 1.84, *p*_2_ = 0.078 > 0.05/8, Cohen’s *d* = 0.27, *CI* = [0.38, 6.75], respectively]. The accuracy of the A1_F2B1 condition (80%) was significantly lower than that of the A1_F1B1 (94%) and A1_F2B2 (94%) conditions [*t*_1_(26) = 4.32, *p_1_* = 0.000 < 0.001/8, Cohen’s *d* = 0.98, *CI* = [7.65, 21.54] and *t*_2_(26) = 4.35, *p_2_* = 0.000 < 0.001/8, Cohen’s *d* = 0.95, *CI* = [7.51, 20.94], respectively]. The accuracy of the A2_F2B1 condition (82%) was significantly lower than that of the A2_F1B1 (95%) and A2_F2B2 (94%) conditions [*t*_1_(26) = 4.35, *p_1_* = 0.000 < 0.001/8, Cohen’*s d* = 0.86, *CI* = [6.50, 18.17] and *t*_2_(26) = 4.68, *p_2_* = 0.000 < 0.001/8, Cohen’s *d* = 0.87, *CI* = [6.64, 17.06], respectively]. These results indicated that when the number of visual stimuli did not match the number of auditory stimuli (the F1B2 and F2B1 conditions), the auditory dominance effect occurred, that is, the number of auditory stimuli affected the judgment of the number of visual stimuli.

### Young Adult Group Analysis (Mean Age = 22)

To further investigate the potential interaction between illusion type and the number of repeated auditory stimuli in young adults, we performed a 2 (number of repeated auditory stimuli: one vs. two) × 2 (illusion type: fission illusion vs. fusion illusion) repeated-measures ANOVA for the young adults group. The main effect of the number of repeated auditory stimuli was significant, *F*(1,25) = 52.77, *p* < 0.001, η*^2^* = 0.68, indicating that the accuracy under the two repeated stimuli condition (79%) was significantly greater than that under the one repeated stimulus condition (68%). The main effect of illusion type was not significant, *F*(1,25) = 1.59, *p* = 0.22, η*^2^* = 0.06. The interaction effect between illusion type and the number of repeated auditory stimuli was not significant, *F* < 1, which meant that the one and two repeated stimuli conditions showed no significant difference under the fission and fusion illusions. To test the influence of the number of repeated auditory stimuli on fission and fusion illusions, we also performed paired-sample *t* tests on the accuracy between A1_F1B2 and A2_F1B2 and between A1_F2B1 and A2_F2B1. The accuracy under the A1F1B2 condition (75%) was significantly less than that under the A2_F1B2 condition (83%), *t*(25) = 6.69, *p* < 0.001, Cohen’*s d* = 0.56, *CI* = [8.12, 15.34]. The accuracy under the A1_F2B1 condition was significantly greater than that of the A2_F2B1 condition, *t*(25) = 3.72, *p* = 0.01, Cohen’s *d* = 0.40, *CI* = [4.96, 17.27]. These results indicated that the repetition of an auditory-based modality could affect not only the fission illusion (F1B2) but also the fusion illusion (F2B1).

**FIGURE 3:**
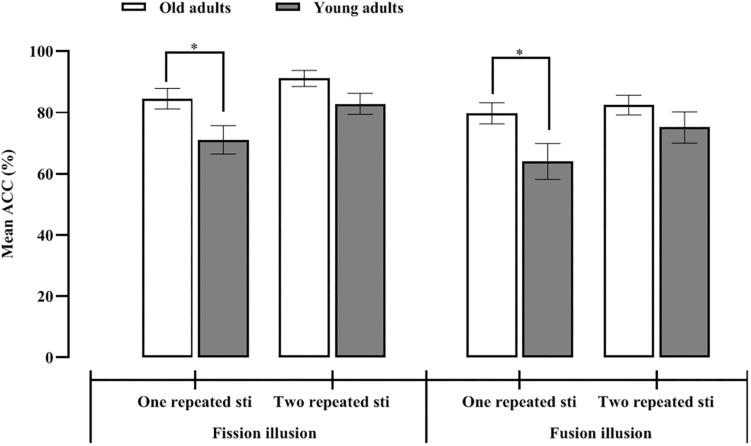
Mean proportion of illusory responses for the one and two repeated auditory stimulus conditions in the young and old adults for the fission **(left)** and fusion **(right)** illusions.

Figure 2 shows that regardless of whether one or two repeated auditory stimuli was presented, the accuracy of the participants under the F1B1 and F2B2 conditions was significantly higher than that under the other conditions; that is, when the number of visual stimuli were consistent with the number of accompanying auditory stimuli, the participants were able to make more accurate judgments that were not affected by the number of repeated stimuli. To prove that the SiFI was present in the young adults, we performed eight paired-sample *t* tests with Bonferroni correction on the following experimental conditions. The accuracy of the A1_F1B2 condition (71%) was significantly lower than that of the A1_F1B1 (97%) and A1_F2B2 conditions (*M* = 95%, *SD* = 5.91) [*t*_1_ (25) = 5.86, *p_1_* = 0.000 < 0.001/8, Cohen’s *d* = 1.50, *CI* = [16.72, 34.82] and *t*_2_(25) = 4.75, *p_2_* = 0.000 < 0.001/8, Cohen’s *d* = 1.38, *CI* = [13.45, 34.08], respectively]. The accuracy of the A2_F1B2 condition (83%) was lower than that of the A2_F1B1 (96%) and A2_F2B2 (95%) conditions [*t*_1_(25) = 4.20, *p*_1_ = 0.000 < 0.001/8, Cohen’s *d* = 0.95, *CI* = [6.56, 19.21] and *t*_2_(25) = 3.33, *p*_2_ = 0.003 < 0.05/8, Cohen’s *d* = 0.92, *CI* = [4.72, 20.05], respectively]. The accuracy of the A1_F2B1 condition (64%) was significantly lower than that of the A1_F1B1 (97%) and A1_F2B2 (95%) conditions [*t*_1_(25) = 5.34, *p_1_* = 0.000 < 0.001/8, Cohen’s *d* = 1.53, *CI* = [20.18, 45.52] and *t*_2_(25) = 5.85, *p_2_* = 0.000 < 0.001/8, Cohen’s *d* = 1.43, *CI* = [19.98, 41.72], respectively]. The accuracy of the A2_F2B1 condition (75%) was significantly lower than that of the A2_F1B1 (96%) and A2_F2B2 (95%) conditions [*t*_1_(25) = 3.71, *p_1_* = 0.001 < 0.01/8, Cohen’s *d* = 1.08, *CI* = [9.15, 32.00] and *t*_2_(25) = 4.84, *p_2_* = 0.000 < 0.01/8, Cohen’s *d* = 1.05, *CI* = [11.52, 28.63], respectively] (see Figure 3). These results indicated that when the number of visual stimuli did not match the number of auditory stimuli (F1B2 and F2B1 conditions), the auditory dominance effect occurred, that is, the number of auditory stimuli affected the judgment of the number of visual stimuli.

### Fission Illusion Analysis of the Two Groups

To investigate the differences in the effects of age-related RS on SiFI under the fission illusion, 2 (participant group: old vs. young adults) × 2 (number of repeated auditory stimuli: one vs. two) repeated-measures ANOVA under the fission illusion condition (F1B2) was performed. The main effect of the number of repeated auditory stimuli was significant, *F*(1,51) = 62.89, *p* < 0.001, η*^2^* = 0.55, indicating that the accuracy under two repeated stimuli (87%) was significantly greater than that under one repeated stimulus (78%). The main effect of participant group was significant, *F*(1,51) = 4.86, *p* = 0.032, η*^2^* = 0.09, indicating that the accuracy of the old adults (88%) was significantly greater than that of the young adults (77%). The interaction effect between the participant group and the number of repeated auditory stimuli was significant, *F*(1,51) = 5.04, *p* = 0.029, η*^2^* = 0.09, which meant that the old and young adults showed different performances under the one and two repeated stimuli conditions. Simple effect analysis indicated that under the one auditory repeated stimulus condition, the accuracy of the old adults (85%) was significantly greater than that of young adults (71%), *t*(51) = 2.36, *p* = 0.022, Cohen’s *d* = 0.69, *CI* = [1.99, 24.90]. In the two auditory repeated stimuli condition, there was no significant difference in accuracy between the two groups (*M_*old*_* = 91%, *M_*young*_* = 83%), *t*(51) = 1.92, *p* = 0.061, Cohen’s *d* = 0.53, *CI* = [−0.38, 16.92] (see Figure 3). Therefore, regardless of whether one or two auditory stimuli were presented, the accuracy of the old adults was greater than that of young adults, which indicated that the fission illusion is less prominent in the old adults.

### Fusion Illusion Analysis of the Two Groups

To investigate differences in the effects of age-related RS on SiFI under the fusion illusion, 2 (participant group: old vs. young adults) × 2 (number of repeated auditory stimuli: one vs. two) repeated-measures ANOVA was performed for the young and old adults under the fusion illusion condition (F2B1). The main effect of the number of repeated auditory stimuli was significant, *F*(1,51) = 17.37, *p* < 0.001, η*^2^* = 0.25, indicating that the accuracy under the two repeated stimuli condition (83%) was significantly greater than that under the one repeated stimulus condition (72%). The main effect of participant group was marginally significant, *F*(1,51) = 3.48, *p* = 0.068, η*^2^* = 0.06, which meant that the accuracy of the old adults (81%) was not significantly different from that of the young adults (70%). The interaction effect between participant groups and the number of repeated auditory stimuli was significant, *F*(1,51) = 6.53, *p* = 0.014, η*^2^* = 0.11, which meant that the old and young adults showed different performances under the one and two repeated stimuli conditions. Simple effect analysis showed that in the one repeated auditory stimulus condition, the accuracy of the old adults (80%) was significantly greater than that of the young adults (64%), *t*(51) = 2.33, *p* = 0.024, Cohen’s *d* = 0.63, *CI* = [2.17, 29.31]. In the two auditory repeated stimuli condition, there was no significant difference in accuracy between the two groups (*M_*old*_* = 82%, *M_*young*_* = 75%), *t*(51) = 1.22, *p* = 0.23, Cohen’s *d* = 0.22, *CI* = [−4.74, 19.33] (see Figure 3). These results indicated that regardless of the number of auditory stimuli (one or two), the accuracy was greater in the old group, suggesting that the fusion illusion was less common in the old adults. Furthermore, regardless of whether the number of repeated auditory stimuli was inconsistent or consistent with the number of flashes, the accuracy of the old adults was greater than that of the young adults.

## Discussion

A lower perceptual sensitivity could be the cause of a higher SiFI and could therefore be a measure of susceptibility to the illusions (McCormick and Mamassian, 2008; Kumpik et al., 2014). This, together with the RS, could influence the intensity of neural activity in the perceptual sensitivity to the stimuli (Grill-Spector and Malach, 2001). Therefore, in the present study, based on the classical SiFI paradigm (Shams et al., 2000, 2002), we added repeated auditory stimuli prior to the presentation audiovisual stimuli to investigate whether the bottom-up factor of RS affected the SiFI with aging. The results replicated previous findings that showed that the illusions were different between the old and young adults; that is, the illusion effect was lower in old adults than in young adults, regardless of the type of illusion (fusion or fission). Moreover, the illusion effect was lower in old adults than in young adults only for the one repeated auditory stimulus condition. In addition, for the fission illusion, the illusion effect was higher for the one repeated auditory stimulus condition than the two repeated stimuli condition in both the old and young adults. These results indicated that the old adults were more affected by the auditory RS than the young adults, that is, the perceptual sensitivity of the auditory stimuli was lower in the old adults, resulting in their being more dependent on visual stimuli. Therefore, the illusion effects were lower.

### Age-Related Differences Affected the SiFI

The classical SiFI effect (Shams et al., 2000) has been shown in both old and young adults both in the present study and in previous studies. Our results showed that under both the one and two repeated stimuli conditions, the illusion effect in the old adults was always lower than that in young adults, which is not consistent with studies on age-related differences in multisensory integration. Many studies have shown that old adults were more susceptible to the SiFI, showing a larger magnitude of the illusion (Setti et al., 2011; DeLoss et al., 2013; McGovern et al., 2014). However, some studies do suggest that the RT in old adults is longer under multisensory integration but does not reflect the magnitude of illusion (Stephen et al., 2010). The reason for the contradiction with previous studies lies in the effect of RS on the SiFI in the present study. Old adults were more affected by the auditory RS than young adults, resulting in a lower magnitude of the illusion effect (either fusion or fission illusion) in the old adults. We conducted a supplementary experiment (see “Supplementary Materials”) in which no auditory repetition was performed. The results showed that under the fission illusion condition, there was no difference in the magnitude of the illusion effect between the old and young adults, however, under the fusion illusion condition, the magnitude of the illusion effect in the old adults was significantly greater than that in the young adults. The pattern of results in the present study are similar to those of Setti et al. (2011) and McGovern et al. (2014). Setti et al. (2011) found that when the SOA was 70 ms, the magnitude of the fission illusion effect between healthy old and young adults was almost equal. In McGovern et al. (2014), when the SOA was 50 ms, the pattern of results between old and young adults was the same as that seen in the supplementary experiment in the present study. However, when we added repeated stimuli, the illusion effect in the adults was significantly lower than that in the young adults, suggesting that the old adults were more susceptible to RS, that is, the perceptual sensitivity of auditory stimuli was lower, resulting in their being more dependent on visual stimuli.

### The Effects of Repetition Suppression on SiFI

Under the F1B2 and F2B1 conditions, there were significant differences between no, one and two repeated stimuli, indicating that repeated stimuli could always significantly improve the accuracy of the participants. The present study confirmed that RS, a bottom-up factor, could reduce the SiFI effect steadily. Repeated stimuli improve the visual system’s ability to process the environment, and the sensitivity of neurons in the retina or visual cortex decreases after adaptation (Kohn, 2007; Wark et al., 2007), which indicates that the repeated stimuli could change perceptual sensitivity. RS occurs across multiple time scales in multiple brain regions and is found for both low-level properties (e.g., color, motion) and high-level perceptual categories, such as faces (Grill-Spector et al., 2006). Based on previous studies, when repeated auditory stimuli were presented with the same as the subsequent auditory stimuli, the repeated stimuli could affect the processing of and decrease the neural activity induced by the subsequent auditory stimuli (Lanting et al., 2013). Similarly, some studies also indicated that changes in neural activity could lead to changes in the effect of the flash illusion. For example, a study found that brain polarization was effective in modulating SiFI perception in humans by receiving anodal, cathodal, or sham tDCS (2 mA, 8 min) in the occipital, temporal, or posterior parietal cortices (Bolognini et al., 2011). Furthermore, some studies have shown that under certain conditions, inferences regarding the perceptual nature of the illusory flash can be made (McCormick and Mamassian, 2008). Such studies have suggested that a higher SiFI effect corresponds to a lower sensitivity; the lower perceptual sensitivity, calculated by d’, can be considered a measure of susceptibility to the illusion (McCormick and Mamassian, 2008; Kumpik et al., 2014). In the present study, the RS of auditory stimuli decreased the perceptual sensitivity of auditory stimuli, which resulting in lower auditory dominance. Thus, under the fission and fusion illusion conditions, repeated stimuli could always significantly reduce the illusion effects. Mostly importantly, we also found that the effects of RS on SiFI vary between old and young adults. Old adults were more susceptible to RS on the effects of the SiFI, which further confirmed our hypothesis that RS could reduce perceptual sensitivity, explaining the different SiFI effects.

### The Effects of Repetition Suppression on SiFI With Aging

We found that under the F1B2 and F2B1 conditions, two repeated stimuli could always significantly improve the accuracy of the participants compared to only one repeated stimulus. First, RS has been shown to affect the SiFI, so there were differences between one and two repeated stimuli. Second, the results indicated that the effect of RS on perceptual sensitivity is influenced by the duration and number of repeated stimuli. Grill-Spector and Malach (2001) found that the signal intensity in the lateral occipital cortex (LOC) of the visual system decreased with an increase in the number of repeated stimuli and decreased the fastest for the first 3–4 repeated stimuli (Grill-Spector and Malach, 2001). Meanwhile, when sound stimuli were presented repeatedly, the amplitude of the N1 component induced by the first sound stimulus was the largest, and the N1 induced by the subsequent sound stimulus was significantly reduced (Ritter et al., 1968; Fruhstorfer et al., 1970; Fruhstorfer, 1971; Budd et al., 1998; Okamoto and Kakigi, 2014). Therefore, auditory repetition suppression is influenced by the duration and number of repeated stimuli, and within a certain range, the longer the duration and the greater the number of stimuli, the more that the neural activity decreases. The present study suggested that two repeated stimuli could reduce perceptual sensitivity, resulting in lower illusion effects than those from one repeated stimulus. In addition, age-related differences in the illusion effects only occurred for the one repeated stimulus condition: the accuracy in old adults was significantly greater than that of young adults, but this was not the case for the two repeated stimulus condition. The reason for this result may be that under the one repeated stimulus condition, the old and young adults showed different perceptual sensitivities, with the old adults having lower perceptual sensitivities to auditory stimuli than the young adults, resulting in lower illusion effects. However, the threshold for perceptual sensitivity was reached under the two repeated stimuli condition, so there was no difference between the two groups.

Repeated auditory stimuli affected the fission and fusion illusions in old and young adults differently. For the fission illusion, both age groups were greatly affected by the repeated auditory stimuli, showing that there was a significant difference between the A1_F1B2 and A2_F1B2 conditions. However, for the fusion illusion, the old adults were not affected by the repeated auditory stimuli, while the young adults were. This showed that the effect of RS on SiFI varied with age, so the fission and fusion illusions performed differently between the groups. These results were consistent with previous studies showing that the fission illusion was more stable and prone to be influenced by bottom-up factors, leading to varying degrees of changes in the illusion effects (Shams et al., 2000; Wozny et al., 2008; Bolognini et al., 2011). Setti et al. (2011) found that old adults were susceptible to the SiFI over a much wider range of cross-modal SOA than young adults, indicating that although the integration time window of the fission illusion in the old adults was longer, there was a stable fission illusion effect in old adults. In the present study, repeated auditory processing had a great influence on the degree of the fission illusion effect but not on the degree of the fusion illusion effect. We speculated that the reason why the fusion illusion was not affected was that there was a less clear or more complex effect of auditory repetition on the fusion illusion.

Although the present study suggested that the bottom-up factor of RS could affect the SiFI with differences between old and young adults, to date there is no consensus as to how the adaptation effect (a similar effect as the RS) affects the SiFI: that is, how the effect of the SiFI changes when the duration of the repeated stimuli is varied from a few milliseconds to a few minutes up to several days. In addition, in the present study, only auditory modal stimulation was used, but visual modal stimulation was not considered as the repetitive stimuli. Additionally, because the temporal window of integration (TWI) is larger in old adults than in young adults, future studies aiming to investigate how RS affects the SiFI differently between old and young adults should take the TWI into account to ensure that the classical illusions are similar.

## Conclusion

In summary, the present study showed that the SiFI effect in multisensory integration was regulated by the repetition suppression (RS) effect, which might be influenced by perceptual sensitivity. The RS effect on the SiFI in old adults was larger than that in young adults, which indicates that the multisensory perceptual sensitivity of old adults is stronger, leading to a weakened SiFI effect. In addition, the present results suggested that the bottom-up factor of RS could affect the SiFI differently between the old and young adults.

## Data Availability Statement

The raw data supporting the conclusion of this article will be made available by the authors, without undue reservation, to any qualified researcher.

## Ethics Statement

Experiments were performed in accordance with the Declaration of Helsinki and approved by the Ethical Committee of Soochow University.

## Author Contributions

AW and MZ were responsible for the experimental design, interpretation of the data and revising the manuscript. YS, XL, and BL were responsible for the data analysis. YS, XL, and CS-S drafted the manuscript.

## Conflict of Interest

The authors declare that the research was conducted in the absence of any commercial or financial relationships that could be construed as a potential conflict of interest.
