# Supplementary material for: Effects of Repetition Suppression on Sound Induced Flash Illusion With Aging
Source: Front Psychol. 2020 Feb 21;11:216. doi: 10.3389/fpsyg.2020.00216 (PMC7047336; doi:10.3389/fpsyg.2020.00216)
Supplement: Supplementary file 1 [file Data_Sheet_1.doc]

**Supplementary Information**

**Supplementary experiment: Classical SiFI paradigm between old and young adults**

**1 Materials and Methods**

**1.1 [Participants](https://brill.com/view/journals/msr/31/8/article-p729_2.xml#d4980653e357)**

The young adult group included 23 college students (8 males and 15 females), between 18 and 26 years old. The old adult group included 35 people (10 males and 25 females) between 60 and 74 years old; these were not the same groups as the participants in the main experiment. All participants were naive to the experimental protocols and were paid for their participation in the experiment. All participants were rescreened for self-reported eye diseases, neurological disorders (e.g., Alzheimer’s disease, Parkinson’s disease, stroke), and any significant hearing loss. All participants gave written informed consent following the standard of the Declaration of Helsinki. The study was approved by the Ethics Committee of the Department of Psychology, Soochow University.

**1.2 Stimulus and Apparatus**

All stimuli and apparatuses were identical to those mentioned in the main experiment.

**1.3 Experimental design and Procedure**

The experiment had a 2 (participant group: old vs. young adults) × 2 (number of visual flash stimuli: one vs. two) × 2 (number of auditory stimuli: one vs. two) mixed design; the participant group was the between-group variable, and others were the within-group variables. The latter two factors constitute the four experimental conditions (F1B1, F1B2, F2B1, F2B2). The design and procedure were similar to those mentioned in the main experiment. The only difference was that the present experiment did not involve the repeated auditory stimuli. The experiment asked participants to judge the number of visual stimuli and ignore the auditory stimuli. Each participant needed to complete 240 trials (40 trials per block, 6 blocks in total), 60 trials under each experimental condition, and the interval between trials was randomized from 400 ms to 700 ms in steps of 100 ms.

**2 RESULTS**

**2.1 Comprehensive analysis of the two groups**

We performed a 2 (participants group: old vs. young adults) × 2 (illusion type: fission illusion vs. fusion illusion) repeated-measures ANOVA. The main effect of participant group was not significant (*M_old_* = 66%, *M_young_* = 70%), *F* (1, 56) = 1.96, *p* = 0.17, *η^2^* = 0.03. The main effect of illusion type was significant, *F* (1, 56) = 10.98, *p* = 0.002, *η^2^* = 0.16, indicating that the accuracy under the fusion illusion condition (50%) was greater than that under the fission illusion condition (37%). The interaction effect between the participants group and illusion type was significant, *F* (1, 56) = 7.33, *p* = 0.009, *η^2^* = 0.12, which meant that the old and young adults showed different performance under the fission and fusion illusions. Simple effect analysis of the participant group showed that under the fission illusion condition, there was no significant difference between the accuracy of old adults (39%) and the accuracy of young adults (34%), *F* < 1; under the fusion illusion condition, the accuracy of young adults (62%) was significantly greater than the accuracy of old adults (41%), *F* (1, 56) = 7.98, *p* = 0.007. Simple effect analysis for illusion type showed that for old adults, there was no significant difference between the accuracy under the fission illusion condition (41%) and under the fusion illusion condition (39%), *F* (1, 56) = 15.01, *p* < 0.001; for the young adults, the accuracy under the fusion illusion condition (61%) was significantly greater than the accuracy under the fission illusion condition (34%), *F* (1, 56) = 7.98, *p* = 0.007.

**2.2 Combined repeated condition in fission illusion analysis**

To explore the performance of the different age groups under the fission illusion condition, we first conducted one-way ANOVA for three different numbers of auditory repeated stimuli (zero, one, and two) for the old and young adults. For the old adults, the main effect of the number of auditory repeated stimuli was significant, *F* (2, 88) = 55.88, *p* < 0.001, *η^2^* = 0.56. According to the post hoc test, the accuracy under the no auditory repeated stimuli condition was significantly lower than that under the one and two auditory stimuli conditions, *t_1_* (61) = 7.38, *p* < 0.001, Cohen’s *d* = 1.99, *CI* = [33.45, 58.31] and *t_2_* (61) = 8.82, *p* < 0.001, Cohen’s *d* = 1.76, *CI* = [40.53, 64.30], respectively. There were significant differences between the one and two repeated stimuli conditions, *t* (27) = 4.35, *p* < 0.001, Cohen's *d* = 0.3, *CI* = [3.59, 9.55] (see Table 1). For the young adults, the main effect of the number of auditory repeated stimuli was significant, *F* (2, 72) = 32.52, *p* < 0.001, *η^2^* = 0.48. According to the post hoc test, the accuracy under the no auditory repeated stimuli condition was significantly lower than that of the one and two auditory stimuli conditions, *t_1_* (47) = 5.43, *p* < 0.001, Cohen’s *d* = 1.54, *CI* = [23.33, 50.82] and *t_2_* (47) = 8.17, *p* < 0.001, Cohen’s *d* = 2.33, *CI* = [36.79, 60.82], respectively. There were significant differences between the one and two stimuli conditions, *t* (25) = 6.69, *p* < 0.001, Cohen'*s d* = 1.89, *CI* = [8.12, 15.34] (see Table 1). The results showed that the fission illusions in both old and young adults were affected by RS.

Then, we performed a *t* test on the accuracy rate of the F1B2 condition for the old and young adults under the no, one and two repeated auditory stimuli conditions. The results showed that there was no significant difference in the accuracies between the old and young adults when there was no repeated auditory stimulus, *t* < 1; when there was one repeated auditory stimulus, the accuracy of the old adults was significantly greater than that of the young adults, *t* (52) = 2.35, *p* = 0.023, Cohen's *d* = 0.64, *CI* = [1.92, 24.43]; and when there were two repeated auditory stimuli, there was no significant difference between the accuracies of the old and young adults, *t* (52) = 1.89, *p* = 0.064, Cohen's *d* = 0.46, *CI* = [-0.49, 16.52] (see Table 1). The ACC of the old and young adults was not significantly different under the no auditory repeated stimuli condition; however, there was a significant difference under the one and two auditory repeated stimuli conditions, with the ACC of the old adults being significantly greater than that of the young adults. The results indicated that the fission illusion in the old adults was more easily affected by RS, resulting in weaker illusion effects than in young adults.

**2.3 Combined repeated condition in fusion illusion analysis**

To explore the performance of the different age groups under the fusion illusion, we first conducted one-way ANOVA for the three different numbers of repeated auditory stimuli (zero, one, and two) for the old and young adults. For the old adults, the main effect of the number of repeated auditory stimuli was significant, *F* (2, 88) = 32.38, *p* < 0.001, *η^2^* = 0.42. According to the post hoc test, the accuracy of the no auditory repeated stimuli condition was significantly lower than that of the one and two auditory stimuli conditions, *t_1_* (61) = 6.00, *p* < 0.001, Cohen’s *d* = 1.61, *CI* = [25.52, 51.06] and, *t_2_* (61) = 6.51, *p* < 0.001, Cohen’s *d* = 1.77, *CI* = [28.37, 53.50], respectively. There were significant differences between the one and two stimuli conditions, *t* (27) = 1.88, *p* = 0.071, Cohen's *d* = 0.15, *CI* = [-0.25, 5.68] (see Table 1). For the young adults, the main effect of the number of auditory repeated stimuli was significant, *F* (2, 72) = 1.88, *p* = 0.161, *η^2^* = 0.05. According to the post hoc test, the accuracy of the no auditory repeated stimuli condition was not significantly lower than that either the one or two auditory stimuli condition, *t_1_* <1, *t_2_* (47) = 1.94, *p* = 0.058, *Cohen’s d* = 2.33, *CI* = [-27.15, 0.48]. There were significant differences between the one and two stimuli conditions, *t* (25) = 3.72, *p* = 0.01, Cohen's *d* = 1.05, *CI* = [4.96, 17.27] (see Table 2). The results showed that the fusion illusions in both old and young adults were affected by RS.

Then, we performed a *t* test on the accuracy rate of the F2B1 condition for the old and young adults under the no, one and two repeated auditory stimuli conditions. The results showed that there was a significant difference between the old and young adults when there was no repeated auditory stimulus, *t* (56) = 2.82, *p* = 0.007, *Cohen's d* = 0.65, *CI* = [5.91, 34.74]. The accuracy of the old adults was significantly higher than that of the young adults when there was one repeated auditory stimulus, *t* (52) = 2.35, *p* = 0.023, Cohen’s *d* = 0.63, *CI* = [2.25, 28.90]. In the case of two repeated auditory stimuli, there was no significant difference in the accuracy between the groups, *t* (52) = 1.22, *p* = 0.23, *Cohen's d* = 0.22, *CI* = [-4.64, 18.98] (see Table 1). The accuracy was significantly different among the no, one and two auditory repeated stimuli conditions for the old adults but not for the young adults. The results indicated that the fusion illusion in old adults was more easily affected by RS, resulting in a lower illusion effect than in young adults.

Table 1 Average accuracy (%) and standard deviation (SD) under fission and fusion in old and young adults under the conditions of no, one, and two repeated stimuli in the supplementary experiment.

|  | Young adults | | | Old adults | |
| --- | --- | --- | --- | --- | --- |
|  | | Fission illusion | Fusion illusion | Fission illusion | Fusion illusion |
| No repeated sti | | 34±24 | 62±21 | 39±29 | 41±29 |
| One repeated sti | | 71±24 | 64±30 | 85±17 | 80±17 |
| Two repeated sti | | 83±18 | 75±26 | 91±14 | 82±17 |
